# "Drain Dance" technique for thoracoscopic application of Tissuepatch to reduce post-operative air-leak saves time and cost

**DOI:** 10.1186/1749-8090-8-S1-O218

**Published:** 2013-09-11

**Authors:** K Ang, I Oey, S Rathinam

**Affiliations:** 1 Dept of Thoracic Surgery, Glenfield Hospital, Leicester, UK

## Background

Tissuepatch™ is an effective adjunct to reduce post-operative air-leak particularly after lung volume reduction surgery. However, thoracoscopic application of Tissuepatch™ can be time-consuming and challenging despite several modifications to the manufacturer's delivery kit. Wastage of Tissuepatch™ during its application remains a problem due to the accidental contamination and activation of the Tissuepatch™ at the tip of the delivery kit when it comes in contact with blood or tissue. We describe a new "Drain Dance" technique to apply Tissuepatch™ effectively and quickly.

## Method

In brief, we had protected the tip of the delivery kit using a chest drain. This also facilitated the introduction of the delivery kit into the thorax. The chest drain cover was then removed, to allow the elegant unrolling of the Tissuepatch™ inside the chest.

## Results

Using this technique in 10 patients to-date (9 unilateral and 1 bilateral procedures), the Tissuepatch™ could be applied successfully in less than 5 minutes per application. It also reduced the accidental wastage of at least one Tissuepatch™ per case.

## Conclusion

The “Drain Dance” technique therefore ensures the effective thoracoscopic application of Tissuepatch™, avoiding wastage and saving time.

